# Atypical Small Bowel Obstruction Caused by Perforated Cecum: A Case Report

**DOI:** 10.7759/cureus.27863

**Published:** 2022-08-10

**Authors:** Orlando Fleites, Stephanie Pelenyi, Kevin Pena, Frederick Tiesenga, Juaquito Jorge

**Affiliations:** 1 General Surgery, Saint James School of Medicine, Park Ridge, USA; 2 General Surgery, Avalon School of Medicine, Willemstad, CUW; 3 General Surgery, West Suburban Medical Center, Oak Park, USA; 4 General and Bariatric Surgery, West Suburban Hospital, Oak Park, USA

**Keywords:** urgent laparotomy, surgery general, obstipation, cecal perforation, small bowel obstruction

## Abstract

Acute onset of abdominal pain with emesis and lack of stool or flatus is an alarming presentation for possible small bowel obstruction (SBO). SBO should be high on the differential diagnosis due to concomitant signs and symptoms that are highly sensitive in diagnosing SBO. These include diffuse tenderness on palpation of the abdomen, abdominal distention, hypotension, vomiting, and lack of flatus or stool. In this report, we present a 67-year-old African American male, who presented to the emergency department with the above-mentioned signs and symptoms and decreased oral intake for four days, ultimately undergoing surgical exploration to relieve the SBO caused by an idiopathic cecal perforation. This case report calls attention to the decision-making, standard protocol, and surgical intervention of a patient with SBO.

## Introduction

Small bowel obstruction (SBO) accounts for 12-26% of surgical admissions and 20% of emergency surgeries in the United States annually [1]. Intra-abdominal adhesions account for 55-75% of SBOs while malignancies, hernias, irritable bowel diseases, and various causes account for the remainder [2,3]. SBO occurs when there is a mechanical interruption to the flow of intestinal content through the bowel. This interruption causes the small intestine proximal to the obstruction to dilate. Patients with bowel obstruction typically present with nausea, vomiting, abdominal pain, and abdominal distention [4].

A cecal perforation that causes a small bowel obstruction is an extremely rare case. Most cases of cecum perforation have been associated with malignancies in the large bowel [5,6]. Cecal perforation normally tends to be a consequence of large bowel obstruction, due to a fully competent ileocecal valve that does not allow the retrograde flow of content [7]. Bowel perforation of any kind can be secondary to many factors, including inflammation, infection, trauma, invasive procedures, medications, or idiopathic. Perforation of any part of the bowel presents clinical challenges. Approximately 14% of patients have an elevated risk of mortality due to intestinal perforation [8]. 

Clinical management for SBO includes non-operative methods, as well as surgery either laparoscopically or through exploratory laparotomy. The objective of this study is to highlight the importance of proper clinical management to prevent mortality with conventional SBO relief methods regardless of etiology. 

## Case presentation

The case is of a 67-year-old male with a chief complaint of diffuse abdominal pain for the previous four days before hospital admission. The patient had a four-day history of dizziness, nausea, emesis, and no passage of flatus or stool before presenting to the emergency department. Per the patient’s recollection, this was the first time experiencing these symptoms. The patient had no solid food intake since the inception of abdominal pain, except for water due to multiple episodes of postprandial emesis. The patient denied any bloody or bilious emesis. The patient also denied any history of recent travel. 

The patient’s medical history was noncontributory towards SBO or cecal perforation, only stating medical management for hypertension and diabetes mellitus type 2 with occasional alcohol consumption. There was no recent change in his current regimen of medication. On further questioning, the patient denied any prior abdominal surgeries.

On physical examination, the patient was in acute distress and hypotensive with systolic blood pressure in the 50s and tachycardia of 110 beats per minute. Sepsis was initially a differential diagnosis, and two liters of 0.9% normal saline along with broad-spectrum antibiotics were administered with an improvement in the systolic blood pressure to 106 mmHg. The patient was alert and oriented but ill-appearing and slightly febrile with a temperature of 38.1°C (100.58°F). He had a noticeably distended abdomen with diffuse abdominal pain and tenderness to palpation. Auscultation of the abdomen revealed high-pitched bowel sounds. 

**Figure 1:**
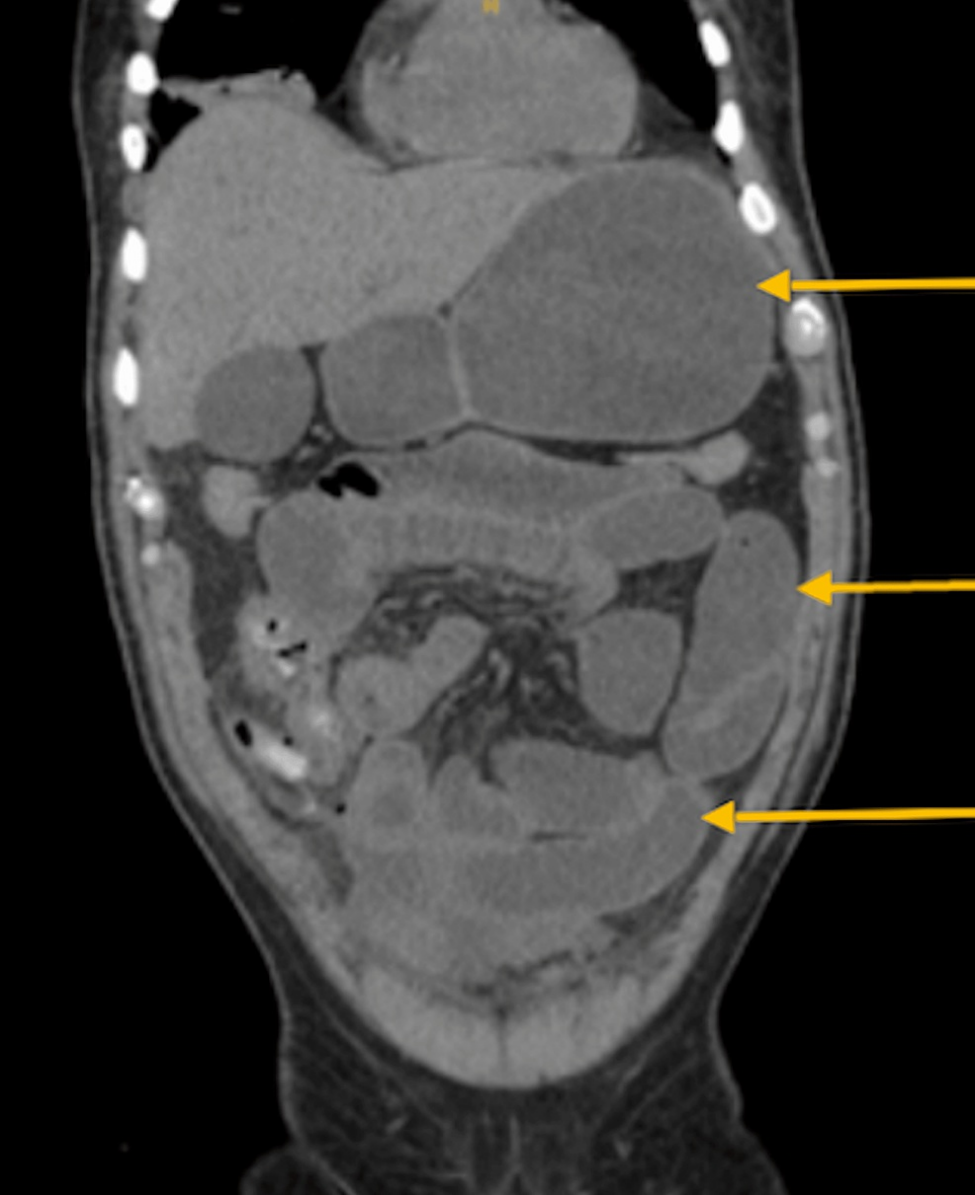
CT obtained on ED presentation, showing evidence of SBO with dilated loops of bowel (yellow arrows) SBO: small bowel obstruction

**Figure 2:**
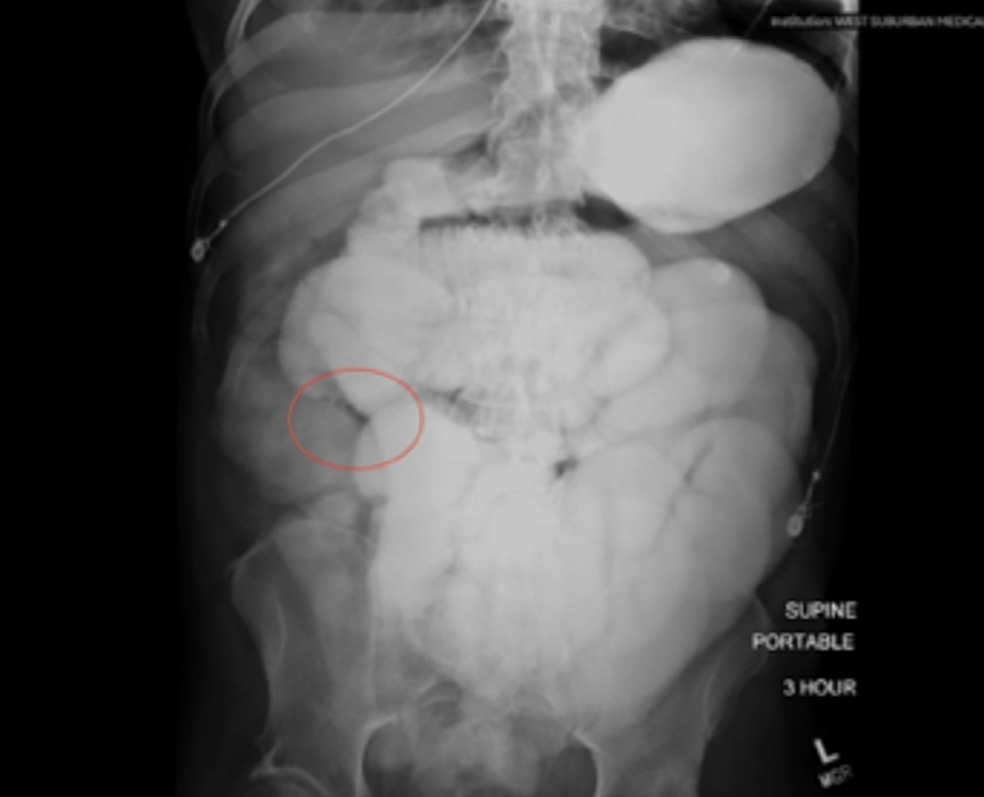
Initial SBFT, showing the SBO transition point (red circle) in the distal portion of the small bowel SBFT: small bowel follow-through; SBO: small bowel obstruction

Initial laboratory workup showed a significant anion gap of 25 mEq/L, leukocyte count of 11,000 x 10^9/L, lactate level below 4 mmol/L, and acute onset renal failure showing a creatinine of 7.3 mg/dL with a BUN of 77 mg/dL. Along with the initial workup of labs, an initial CT was ordered due to the high suspicion of SBO. The initial impression of the CT scan of the abdomen and pelvis showed multifocal airspace opacities throughout the lung bases, fluid in bilateral paracolic gutters, and pelvis likely reactive to the patient's ongoing symptoms. No free air or fluid was noted but small bowel loops were observed to be diffusely dilated (Figure 1) with no clear indication of cecal perforation. These findings were secondary to possible ileus, SBO, or enteritis. Initial non-operative management with nasogastric (NG) tube placement produced a significant amount of output measuring one liter of bilious content. A small bowel follow-through (SBFT) was subsequently ordered less than 24 hours after patient admission to better identify the transition point. In comparison to the previously performed CT scan, the findings of SBFT were compatible with an SBO with its transition point in the most distal portion of the small bowel (Figure 2). 

Initial non-operative/conservative management through the NG tube was ordered upon ED presentation. The patient continued to have diffuse abdominal pain. Confirmed SBO through SBFT and CT scan, the patient's worsening diffuse abdominal pain out of proportion to examination, continued acidosis, tachypnea, and failed decompression of SBO with NG tube were indications for the patient to undergo surgical exploration. Upon entering the peritoneal cavity a considerable amount of malodorous material was identified in the right lower quadrant, which was then cultured. In the right lower quadrant, the transition point was located along the white line of Toldt. The small bowel proximal to this area was matted with purulent material walled off behind the small bowel. Continued exploration determined there was cecal perforation with fecal matter draining through. The curative surgical procedure for this patient was an exploratory laparotomy and a right hemicolectomy with diverting loop ileostomy. The postoperative diagnosis was an SBO due to a idiopathic perforated cecum. 

The patient, post-operatively, was intubated and taken to the intensive care unit due to postoperative pulmonary complications of atelectasis and impairment of spontaneous respiration. One week postoperatively the patient was maintained on ventilator support. It was planned for pulmonary toilet post extubation. Follow-up abdominal and pelvic CT scans were ordered due to a persistently elevated leukocyte count, but no attributable causes were discovered. The patient continued broad-spectrum antibiotics. Roughly three weeks post surgery, the patient began to have optimal ostomy output as well as normal respiratory function with minimal pain. Acute kidney injury was resolved by this point and our patient was eventually discharged with recommendations to follow up as an outpatient. 

## Discussion

An SBO is a disruption of intestinal content through the small intestine by a mechanical source, e.g., stricture, adhesion, or collapsed bowel [4,9,10]. SBO can be classified into two different groups: complete bowel obstruction and partial bowel obstruction. Complete bowel obstruction is when no bowel content flows through the gastrointestinal tract and results in the patient experiencing obstipation, i.e., the lack of flatus or stool. Partial bowel obstruction is when some bowel content can continue through an obstruction in the intestinal tract, with flatus and possible liquid stools reported by the patient [10]. 

Initial treatment for SBOs is dependent on clinical presentation alongside diagnostic evaluations. In nonemergent SBOs, non-operative/conservative management is promptly initiated. Conservative management options, dependent on the patient's well-being, include fluid resuscitation, pain control, restricting oral intake, antibiotics, and NG tube placement [11,12]. The aforementioned non-operative management can resolve certain types of SBOs, the sequelae being bowel decompression. Adhesive SBO is due to intra-abdominal adhesions that form due to the normal healing process post-operatively. Consequently, interruption to the peritoneum from any previous abdominal surgery can lead to the formation of adhesions. Adhesive SBO may be managed nonoperatively if the patient appears clinically fine and the patient's vitals are within normal limits [13]. 

Failed conservative management and a high pretest probability of bowel compromise (bowel ischemia, necrosis, or perforation) upon clinical or radiographic examination should bypass non-operative management and the patient be taken immediately for surgical intervention [12]. A patient with complete bowel obstruction is at increased risk of life-threatening complications due to bowel ischemia and perforation. Complete bowel obstructions can be caused by an incarcerated hernia, adhesions, or tumors requiring immediate surgical intervention [10]. One example is a closed-loop SBO, which requires immediate surgical intervention. Closed-loop SBO occurs when an adhesion obstructs two different parts along a segment of the bowel at one particular point. The closed-loop obstruction can be secondary to an adhesion, internal herniation, or a twisting of the mesentery, which is an indication to bypass conservative management and elect for immediate surgical intervention to relieve the SBO [1]. Immediate surgical intervention should promptly occur to prevent mortality. Ominous symptoms for immediate surgical intervention are peritonitis, abdominal tenderness, pain, distention, obstipation, and vomiting [10].

Imaging modalities are vital in the medical management of SBO. CT scans continue to be the golden standard imaging modality to confirm SBO; although, in this case, it was during surgical exploration that perforation of the cecum was definitively determined [14]. The imaging modalities with the highest sensitivities to SBO are abdominal x-ray, SBFT, and CT scans. SBFT is employed to assess the location and degree of SBO. SBFT is implemented with the oral intake of Gastrografin, a water-soluble contrast that allows for a series of x-rays to determine the passage of the contrast through the patient's intestines [15]. Imaging via a CT scan is employed to visualize the anatomical location of the obstruction and assess for other related complications [10,16]. A complete bowel obstruction on imaging will show an absence of air or fluid in the distal small bowel past the transition point and the absence of air in the large bowel. Specific radiographic findings such as pneumo-peritoneum, bowel ischemia, or necrosis along with an unwell patient require immediate surgical intervention [12,17].

In lieu of commonly seen SBO etiologies, this case presented with an idiopathic perforated cecum causing an SBO. No large bowel obstruction was found during exploratory laparotomy, which is the leading cause of cecal perforation [5,6,18]. The patient's preoperative obstipation and lack of improvement after conservative management were indications for surgical intervention. While literature reviews maintain a consensus on the predominant causes of cecal perforation and SBO, this case highlights that the standard of care for SBO promptly relieves a patient suffering from a rare or any cause of SBO. It is still imperative to make a timely decision in the medical care of a patient suffering from this condition. 

## Conclusions

An SBO caused by a perforated cecum is a medical anomaly. There are no previously documented cases of this nature. Our case presents an anomaly to the typical etiological course of SBO. This patient also lacked common etiological factors of cecal perforation such as large bowel obstruction, medications, or trauma. Furthermore, this case reaffirms the importance of imaging modalities in diagnosing an SBO due to bowel perforation. CT scan with contrast is the gold standard in confirming SBO while also assessing for any leakage of content from the intestine. Other imaging modalities, such as small bowel follow-through, aid in determining the extent of the SBO. Ultimately, exploratory laparoscopy continues to be the curative intervention of choice for SBO that has failed non-operative management.
